# Low-Cost Ultra-Wide Genotyping Using Roche/454 Pyrosequencing for Surveillance of HIV Drug Resistance

**DOI:** 10.1371/journal.pone.0036494

**Published:** 2012-05-04

**Authors:** Dawn M. Dudley, Emily N. Chin, Benjamin N. Bimber, Sabri S. Sanabani, Leandro F. Tarosso, Priscilla R. Costa, Mariana M. Sauer, Esper G. Kallas, David H. O.’Connor

**Affiliations:** 1 Department of Pathology and Laboratory Medicine, University of Wisconsin-Madison, Madison, Wisconsin, United States of America; 2 Department of Cellular and Molecular Biology, University of Wisconsin-Madison, Madison, Wisconsin, United States of America; 3 Wisconsin National Primate Research Center, University of Wisconsin-Madison, Madison, Wisconsin, United States of America; 4 São Paulo Institute of Tropical Medicine, University of São Paulo, São Paulo, Brazil; 5 Division of Clinical Immunology and Allergy, University of São Paulo, São Paulo, Brazil; University of Hawaii Manoa, United States of America

## Abstract

**Background:**

Great efforts have been made to increase accessibility of HIV antiretroviral therapy (ART) in low and middle-income countries. The threat of wide-scale emergence of drug resistance could severely hamper ART scale-up efforts. Population-based surveillance of transmitted HIV drug resistance ensures the use of appropriate first-line regimens to maximize efficacy of ART programs where drug options are limited. However, traditional HIV genotyping is extremely expensive, providing a cost barrier to wide-scale and frequent HIV drug resistance surveillance.

**Methods/Results:**

We have developed a low-cost laboratory-scale next-generation sequencing-based genotyping method to monitor drug resistance. We designed primers specifically to amplify protease and reverse transcriptase from Brazilian HIV subtypes and developed a multiplexing scheme using multiplex identifier tags to minimize cost while providing more robust data than traditional genotyping techniques. Using this approach, we characterized drug resistance from plasma in 81 HIV infected individuals collected in São Paulo, Brazil. We describe the complexities of analyzing next-generation sequencing data and present a simplified open-source workflow to analyze drug resistance data. From this data, we identified drug resistance mutations in 20% of treatment naïve individuals in our cohort, which is similar to frequencies identified using traditional genotyping in Brazilian patient samples.

**Conclusion:**

The developed ultra-wide sequencing approach described here allows multiplexing of at least 48 patient samples per sequencing run, 4 times more than the current genotyping method. This method is also 4-fold more sensitive (5% minimal detection frequency vs. 20%) at a cost 3–5× less than the traditional Sanger-based genotyping method. Lastly, by using a benchtop next-generation sequencer (Roche/454 GS Junior), this approach can be more easily implemented in low-resource settings. This data provides proof-of-concept that next-generation HIV drug resistance genotyping is a feasible and low-cost alternative to current genotyping methods and may be particularly beneficial for in-country surveillance of transmitted drug resistance.

## Introduction

Availability of antiretroviral therapy (ART) is increasing in low and middle-income countries [1]. There is mounting evidence suggesting that transmitted drug resistance increases over time as ART use increases [2]–[4]. For example, in Kampala, Uganda, a massive scale-up of ART was initiated in the year 2000 and a small survey performed in 2006–2007 suggested no detection of transmitted drug resistance [2]. Another survey performed in Kampala between 2009 and 2010 showed a prevalence of transmitted drug resistance at 8.6%, suggesting that while this resistance may not immediately arise after scale-up, over time it increases in prevalence. Transmitted drug resistance may thwart current efforts to scale-up treatment in low and middle-income settings where few treatment options are available. It is highly recommended by the World Health Organization (WHO) that surveillance of drug resistance occur in conjunction with scale-up efforts to ensure appropriate first-line therapy is offered relative to the resistance that exists [5]. It is believed that surveillance will maximize the utility of first-line therapy and help minimize the cost of providing ART thereby sustaining current antiretroviral drug programs. This is particularly important as treatment guidelines now recommend earlier start of ART, prolonging the period of time individuals are taking antiretroviral drugs, and increasing the opportunity for drug resistance to develop and transmit [6]. However, drug resistance surveillance remains highly expensive and mostly unavailable in many limited resource settings.

We have developed a method using the next-generation Roche/454 sequencing platform to monitor HIV drug resistance through genotyping. By coupling multiplexing together with a lower-cost laboratory-scale next-generation sequencer (Roche/454 GS Junior), we reduce the cost of drug resistance surveillance by 3–5-fold allowing its implementation in resource-limited settings. In addition, because next-generation sequencing is clonal in nature, it provides increased sensitivity over traditional Sanger-based sequencing that will enable future work to understand the dynamics of the emergence of drug resistance within a population. We refer to our approach as “ultra-wide” drug resistance testing because the large number of sequence reads obtained in a single Roche/454 pyrosequencing run can be applied across at least 48 different patient samples. This is different than the more traditional application of sequencing a single patient sample to study HIV in an “ultra-deep” manner. 48 samples is also four times larger than the number of samples that can be simultaneously sequenced using traditional Sanger-based HIV drug resistance genotyping.

Here we present a proof-of-principle study using our Roche/454 pyrosequencing approach to study drug resistance in a cohort of HIV-positive individuals enrolled in a study through the University of São Paulo in Brazil. We obtained samples from 81 HIV-infected individuals either exposed or not exposed to antiretroviral therapy. We designed primers to amplify protease and the first 735 nucleotides of reverse transcriptase to encompass mutations identified through the Stanford HIV drug resistance database and the WHO drug resistance surveillance list. We optimized PCR strategies to amplify HIV from Brazil (primarily subtype B) with a range of viral loads. After multiplexing up to 48 patient samples/sequencing run and analyzing the data through an open-source in-house analysis pipeline, we identified drug resistance mutations in 13/70 (18.5%) of samples in our cohort. When considering only the treatment-naïve samples, 10/50 (20%) of HIV+ individuals harbored one or more drug resistance mutations. Altogether, we show the feasibility of multiplexing 48 patient samples together in a single Roche/454 GS Junior pyrosequencing run to obtain high quality and sensitive HIV drug resistance genotyping information. Through this multiplexing approach, the sequencing cost is reduced to USD $20/sample, which is 3–5× less expensive than traditional Sanger-based sequencing methods performed as part of either in-house or commercial kits. This approach is adaptable to different HIV subtypes and can be scaled up to accommodate larger surveillance efforts.

## Methods

### Ethics Statement

The subjects used in this study provided informed written consent. The protocol and consent form for phlebotomy were approved by the ethics committees at the University of São Paulo (USP) and the Federal University of São Paulo. The collected samples were de-identified and sent to the University of Wisconsin-Madison for sequencing and met the criteria for IRB exemption from the University of Wisconsin health sciences minimal risk IRB, protocol M-2008-1167.

### Study Subjects

Eighty-one patient samples were selected to include in this study from the Serologic Testing Algorithm for Recent HIV Seroconversion (STAHRS) cohort [7]. This cohort, mostly constituted by men who have sex with men recently infected by Clade B strains, continues to collect samples over time for multiple years [8]. 55 samples were collected between 1998 and 2003 from patients prior to antiretroviral treatment (ART). The mean viral load of these samples was 100,547 copies/ml with a standard deviation of 129,646 copies/ml. 12 samples were selected from individuals following ART and with no drug failure as measured by undetectable viral loads. The limit of detection of the viral load assay using the Amplicor HIV-1 Monitor test was 400 copies/ml for samples processed before January, 2007, and 50 copies/ml after this date using the Versant HIV-1 RNA 3.0 assay using branched DNA. Lastly, 14 samples were chosen from individuals failing ART, defined as having detectable viral loads, with mean viral loads of 10,221 copies/ml and a standard deviation of 20,519 copies/ml. Most patients on ART received a standard first-line therapy regimen including efavirenz (non-nucleoside reverse transcriptase inhibitor (NNRTI)), lamivudine (nucleoside reverse transcriptase inhibitor (NRTI)), and zidovudine (NRTI). Two patients (patient ID 2008 and 1015) were taking a combination of zidovudine, lamivudine, lopinavir, and ritonavir and one patient (patient ID 2039) was taking a combination of zidovudine, lamivudine, and atazanavir boosted with ritonavir at the time of post-treatment sample collection. Samples collected after the start of antiretroviral therapy were collected between 2003 and 2007.

### Sample Preparation and PCR Amplification

**Figure 1:**
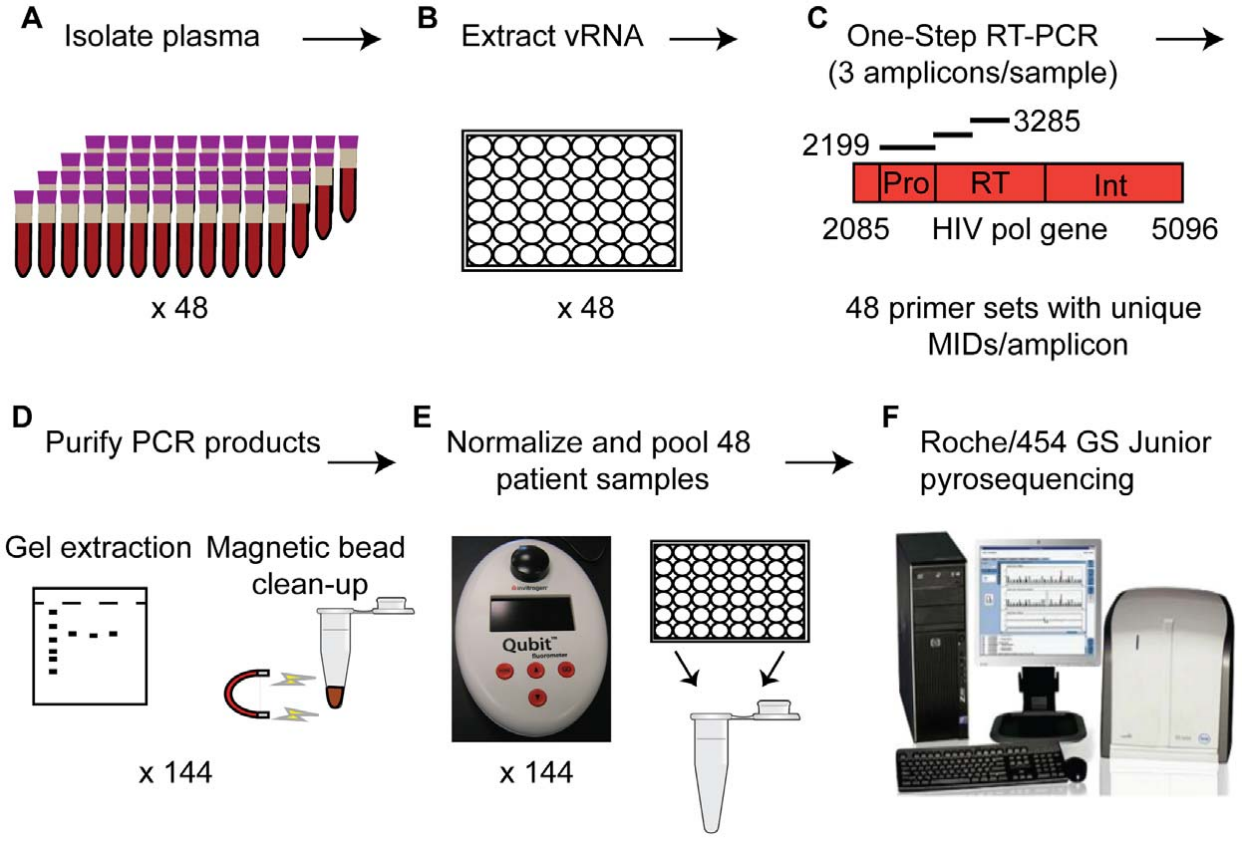
Schematic representation of the sample preparation for ultra-wide HIV drug resistance genotyping using Roche/454 pyrosequencing. A.) Plasma is isolated from 48 patient samples using centrifugation. B.) Viral RNA is extracted from ∼1 ml of plasma from each sample. C.) One-step RT-PCR is used to reverse-transcribe and PCR-amplify 3 amplicons spanning the HIV pol gene from each sample as shown. Each sample is amplified with primers containing a unique multiplex identifier (MID) tag (1–48). D.) PCR products are gel purified and purified further using size exclusion magnetic beads. E.) Purified samples are quantitated and pooled together at equimolar ratios for a total of 144 amplicons/pool. F.) Each pool is subjected to emPCR followed by pyrosequencing on the Roche/454 GS Junior.

A schematic representing the major steps to prepare samples for drug resistance genotyping using the method described in this paper is presented in Figure 1. Plasma from each sample was centrifuged at 14,000 × rpm at 4°C for 1 hour to concentrate virus for viral RNA extraction using the QIAamp Viral RNA Mini Kit (Qiagen). Viral RNA was subjected to one-step RT-PCR amplification using Superscript III Reverse Transcriptase and High Fidelity Platinum Taq Polymerase (Invitrogen). We designed three primer sets that were used to amplify the entire protease gene (Pro) and the first 735 nt of the reverse transcriptase gene in two halves (RT1 and RT2). Fusion primers containing the Roche 454 titanium amplicon adaptor sequences, multiplex identifier (MID) tags on both forward and reverse primers, and the sequence-specific primer were generated (Integrated DNA technologies). The sequence-specific portions of the primers are as follows: Protease (Pro) forward 5′- AACTCCCTCTCMGAAGCAGGAG-3′ and reverse 5′- CTGGCTTTAATTTTACTGGTA-3′; reverse transcriptase 1 (RT1) forward 5′- AAATTAAAGCCAGGAATGGA-3′ and reverse 5′- TGTCTCAATGTTTGTACTAGGTAT-3′; and reverse transcriptase 2 (RT2) forward 5′-TGGGTGATGCATATTTTTCAG-3′ and reverse 5′-GCACTATAGGCTGTACTGTCC-3′. The full sequences containing MID tags and adaptors for the 48 primer sets used to amplify the samples in this project are presented in Table S1. PCR conditions were as follows: 50°C for 60 min. for reverse transcription; 94°C for 2 min. to activate polymerase; 2 cycles of 94°C for 15 sec., 60°C for 30 sec., and 68°C for 30 sec.; 2 cycles of 94°C for 15 sec., 58°C for 30 sec., and 68°C for 30 sec; 36 cycles of 94°C for 15 sec., 55°C for 30 sec., and 68°C for 30 sec.; and lastly a final 10 min. extension was performed at 68°C.

Samples that did not amplify during a single round of PCR were subjected to nested PCR, but were amplified in a total of 40 cycles between both external and nested PCR amplifications. Viral RNA was amplified using the one-step RT-PCR approach as described above with the following external primer pairs: Protease forward 5′-AGCTTCAGGTTTGGGGARGAG-3′ and reverse 5′-TGTTTAACTTTTGGGCCATCC-3′; RT1 forward 5′-CCCATTAGTCCTATTGAAACTG-3′ and reverse 5′-CTGTGGAAGCACATTGTACTG-3′; RT2 forward 5′-GAATACCACATCCCGCAGG-3′ and reverse 5′-ACTTGCCCAATTCAATTTTCC-3′. The PCR conditions were as follows: 50°C for 60 min. for reverse transcription; 94°C for 2 min. for *taq* activation; 2 cycles of 94°C for 15 sec., 60°C for 30 sec., and 68°C for 30 sec.; 2 cycles of 94°C for 15 sec., 58°C for 30 sec., and 68°C for 30 sec; 16 cycles of 94°C for 15 sec., 55°C for 30 sec., and 68°C for 30 sec.; and lastly a final 10 min. extension was performed at 68°C. 15 µl of the 25 µl RT-PCR product was subjected to nested PCR amplification using the same cycling conditions as the external PCR reaction minus the 50°C reverse transcription step, and using the same fusion primer sets described for the single round amplifications above containing Roche 454 adaptors and MID tags.

### Sample Quantitation and Purification

Following PCR amplification, samples with higher viral loads (>2,000 copies/ml) were subjected to agarose gel electrophoresis and gel purified using a MinElute Gel Extraction Kit (Qiagen) (Figure 1). Samples from individuals with low or undetectable viral load were analyzed on an Agilent Bioanalyzer 2100 high sensitivity DNA chip to verify product size and purity. All PCR samples were purified using an AMPure XP (Agencourt) bead clean-up at a bead:DNA volume ratio of 0.8∶1. Samples were then quantitated using picogreen dye and the Qubit (Invitrogen) fluorometer. PCR products were pooled together at equimolar ratios to prepare for Roche/454 pyrosequencing. A final AMPure bead clean-up was performed on the pooled samples and a final Qubit quantitation of the pool was taken to calculate correct copy numbers for emulsion PCR. Finally, the DNA pool was analyzed on the Agilent Bioanalzyer 2100 to ensure removal of all primer/primer-dimer short products by the AMPure bead clean-up before emulsion PCR.

### Ultra-wide Pyrosequencing

All samples were sequenced as part of two independent Roche/454 GS Junior runs. Emulsion PCR was performed according to Roche/454 manufacturer instructions. The sample libraries were added to the emulsion PCR at a ratio of two molecules per bead. Following the enrichment of the emulsion PCR products, the picotiter plate (PTP) was prepared and 500,000 enriched beads were loaded per the instruction manual provided by Roche/454.

### Data Analysis

Sequence files in a Standard Flowgram Format (SFF) generated following the sequencing runs were analyzed using a custom analysis pipeline. This pipeline utilized Samtools and BioPerl [9], [10]. This pipeline has been made available as a module for LabKey Server, an open-source platform for the management of scientific data (www.labkey.com). The LabKey Sequence Analysis module provides a web-based interface to initiate analyses, manage data, and view results. The source code behind this pipeline is available in a subversion repository (https://hedgehog.fhcrc.org/tor/stedi/trunk/server/customModules/SequenceAnalysis). The pipeline performs three steps: pre-processing of sequence, alignment, and SNP calling. The raw sequence data was processed as follows: sequences were converted to FASTQ format, separated by MID tag, trimmed on the 3′ end based on quality scores (using a sliding window of 10 nt, requiring an average quality score of at least 17 in this window), the Roche/454 amplicon adaptors were trimmed (adaptor A: 5′ CGTATCGCCTCCCTCGCGCCATCAG 3′; adaptor B: 5′CTATGCGCCTTGCCAGCCCGCTCAG 3′), and reads less than 200 bp were discarded. Next, the sequences were aligned to the HXB2 reference strain (GenBank NC_001802) using BWA-SW to create a BAM alignment [11]. All bases of the alignment were evaluated and single nucleotide polymorphisms (SNPs) were called for bases with a quality score above 19 and deletion/insertion polymorphisms were called for bases with a quality score above 25. Importantly, the identity of the associated read was retained for each SNP, which allows the phase of SNPs to be considered. This information allowed amino acid translations to be calculated based on the sequence of each individual read, as opposed to the consensus sequence. The information produced during the analysis process was stored in the LabKey database, from which results were viewed and reports generated. The analysis pipeline reduced the sequence data to lists of SNPs relative to the reference strain that were viewed in both nucleotide and amino acid format. To simplify drug resistance analysis further, one of the reports generated by this pipeline presents a list of the specific amino acid changes that correspond to sites of known drug resistance mutations based on the Stanford HIV drug resistance data base (Stanford HIVDB) matrices. Frequencies of each mutation found in our patient samples as well as the number of sequences representing that amino acid site, adjusted for only high quality sequences, were taken from this drug resistance report. Sequence data including the alignment file (.bam) and translated SNPs (.txt) from this analysis is available at https://ehr.primate.wisc.edu/project/WNPRC/WNPRC_Laboratories/oconnor/public/begin.view?. Lastly, sequences were also visualized using Geneious software (version 5.5.6) to confirm the mutations identified by the analysis pipeline as well as to assess which amplicons contained sequence information. The SFF files containing the sequencing results from the Roche/454 GS Junior were uploaded into Geneious and assembled to the HXB2 reference sequence previously described.

### HIV Plasmid and Clonal HIV Stock Sequencing

To assess the error associated with reverse transcription, PCR amplification and Roche/454 pyrosequencing, we sequenced both an HXB2 HIV plasmid (pHXBn-PLAP-IRES-N+ was obtained through the NIH AIDS research and reference reagent program, Division of AIDS, NIAID, NIH from Dr. Benjamin K. Chen and Dr. David Baltimore) and clonal viral stock. To produce the clonal viral stock, the pHXBnPLAP-IRES-N+ (HXBn) plasmid was transfected into 239T packaging cells using the Xfect Transfection Reagent (Clontech). The supernatant was collected two days after transfection to limit virus production to a single round and the virus was quantitated using the Lenti-X qRT-PCR titration kit (Clontech). Viral RNA was isolated from this supernatant (viral load = 115,000 copies/ml) and subjected to one-step RT-PCR and sequenced using the same protocol that was used to sequence patient samples. Both DNA and viral RNA starting templates are expected to have no SNPs relative to the HXB2 reference sequence (GenBank NC_001802). The plasmid DNA was sequenced using the same protocol as the patient samples without the initial reverse transcription step. In addition, we sequenced the HIV plasmid using a Sanger-based approach as a standard to verify the sequence of the plasmid in the pol gene. This verification revealed two mutations in the pol gene (HXB2 positions 1805 and 2473) of the plasmid that were not expected based on the published map of the vector. These mutations in the plasmid were ignored during analysis. All other SNPs found by pyrosequencing in either the plasmid or the viral stock were considered “false SNPs” that likely resulted from error in the PCR, pyrosequencing, or analysis of the sequences. SNPs with frequencies above the “false SNPs” were considered real changes that do not likely stem from error associated with the sequencing method, from which we established the minimum frequency threshold used in our sequence analysis of patient samples (described below).

## Results

### Determining Error Threshold of the HIV Drug Resistance Genotyping Method

**Figure 2:**
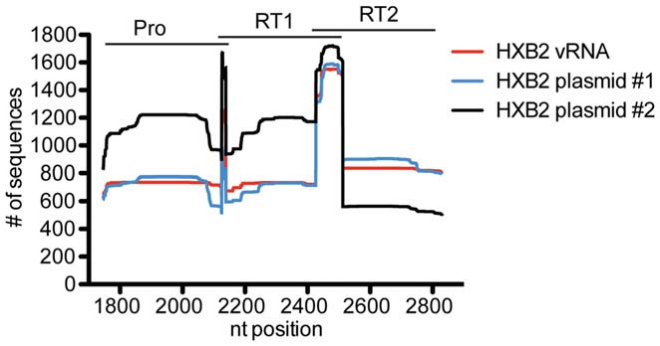
Sequence coverage of three amplicons from a clonal HXB2 viral stock and HXB2 plasmid. The number of sequences from the clonal viral stock (red) or HIV plasmid (blue and black) that aligned to each nucleotide position of the NC_001802 HXB2 HIV reference sequence is shown across the *pol* gene. The HXB2 vRNA clonal viral stock was sequenced under one GS Junior sequencing run, while two independent PCR amplifications were used to sequence the plasmid under two different MID tags in a separate GS Junior sequencing run. Pyrosequencing was performed on three overlapping amplicons with nucleotide positions for each amplicon represented at the top of the graph.

**Figure 3:**
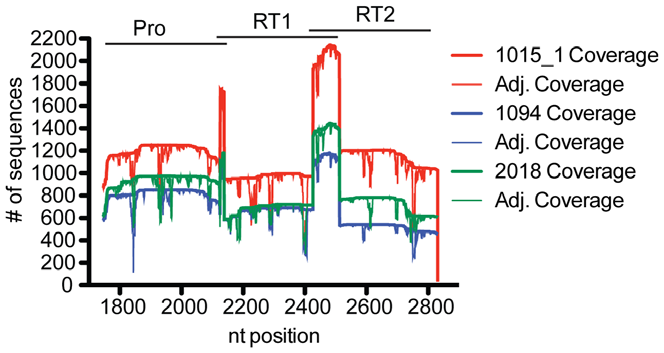
Sequence coverage and adjusted sequence coverage of three representative patient samples. The number of sequences representing each nucleotide position (coverage) from three patient samples sequenced in the same GS Junior run is shown after alignment to the NC_001802 HXB2 HIV reference sequence. Also shown is the number of sequences that align to each nucleotide position after eliminating sequences with low quality scores (adjusted coverage) at each nucleotide position for each patient sample. Pyrosequencing was performed on three overlapping amplicons with nucleotide positions for each amplicon represented at the top of the graph.

To assess the error associated with our sample preparation, sequencing, and analysis methods, we processed and pryosequenced a HXB2 HIV plasmid and a clonal HIV viral stock not expected to have any mutations relative to the HXB2 reference sequence (GenBank NC_001802), as described in the materials and methods. The average depth of sequence coverage per nucleotide position for the clonal virus stock was 826 (+/−212) sequences (Figure 2). As a comparison, the HXBn plasmid was sequenced using the same methods without the reverse transcription step. The plasmid was sequenced from two independent PCR amplifications in one sequence run and was differentiated by a multiplex identifier tag. The average depth of sequence coverage for each MID tag was 826 (+/−223) and 1009 (+/−348) sequences (Figure 2). This sequence coverage is similar to the coverage obtained when sequencing our patient samples (Figure 3).

Analysis of the pyrosequencing results from both the plasmid and viral stock starting material showed no “false” drug resistance mutations above a frequency of 0.71% and in no more than 4 sequence reads (Table 1). Based on this data, we chose a conservative minimal frequency of 5% when assessing our patient samples for drug resistance mutations, ∼7-fold above our predicted error rate. Assessment of the error associated with our sequencing method outside of the drug resistance sites or in changes that don’t create specific drug resistance mutations is provided in Text S1 and Table S2.

**Table 1:** “False” drug resistance mutations found in the HXB2 plasmid or HXB2 viral stock.

HXB2 source^1^	Resistance mutation	Drug class	Frequency (%)	Adjusted coverage	Total reads with mutation
Plasmid 2	M41L	NRTI	0.10	1010.0	1.0
Plasmid 1	K70R	NRTI	0.43	700.0	3.0
Plasmid 1	V106A	NNRTI	0.15	686.0	1.0
Plasmid 1	Y181C	NNRTI	0.11	902.0	1.0
Plasmid 1	M184I/V	NRTI	0.33	902.0	3.0
Plasmid 2	M184I	NRTI	0.18	560.0	1.0
Plasmid 1	Y188H	NNRTI	0.22	896.0	2.0
Plasmid 1	M230L	NNRTI	0.14	705	1.0
Plasmid 1	V32I	Protease	0.13	765.0	1.0
Plasmid 1	M46I/L	Protease	0.71	421.0	3.0
Plasmid 2	M46L	Protease	0.14	707.0	1.0
Virus	I47V	Protease	0.29	695.0	2.0
Plasmid 2	I47V	Protease	0.09	1123.0	2.0
Plasmid 1	I47V	Protease	0.14	721.0	1.0
Plasmid 2	I50V	Protease	0.27	1098.0	3.0
Plasmid 2	I54T/V	Protease	0.40	994.0	4.0
Virus	I54V	Protease	0.16	643.0	1.0
Plasmid 2	L76V	Protease	0.08	1185.0	1.0
Plasmid 2	V82A	Protease	0.17	1203.0	2.0
Virus	V82A	Protease	0.27	736.0	2.0
Plasmid 1	V82A	Protease	0.13	763.0	1.0
Plasmid 1	N88D	Protease	0.28	717.0	2.0
Plasmid 2	N88D/S	Protease	0.35	1142.0	4.0

1 Plasmid 1 and 2 refer to data from two independent experiments used to sequence the same HXBn plasmid. Virus refers to the data from sequencing a viral stock derived from the HXBn plasmid.

### Sequencing Statistics from Patient Samples

Patient samples were sequenced in two independent Roche/454 GS Junior runs (Table 2). After trimming low quality sequences off the ends of the sample reads, the average sequence length that aligned to the reference sequence for each run was 374 bp and 363 bp for runs 1 and 2 respectively. These sizes are slightly smaller than the total amplicon sizes that range from 386 bp–404 bp, indicating near full-length coverage of each sequence from both read directions. The average coverage varied with each sequencing run as well as at each nucleotide site associated with drug resistance mutations. Figure 3 shows coverage of sequences that aligned to HXB2 before (coverage) and after (adjusted coverage) low quality sequences were removed from a few representative samples from the first GS Junior run. In general, coverage spikes correspond to regions of overlap between the primer sets as shown at the top of Figure 3. Coverage dips, which are consistent between samples, typically fall at the ends of homopolymer regions where the sequence quality scores drop due to pyrosequencing chemistry. Adjusted coverage is reported for SNPs found in both the control and patient samples and was used to determine the frequencies reported in Tables 1, 3, and S2.

**Table 2:** The statistics of the two GS Junior runs used to assess the patient samples in this study.

	GS Junior run #1	GS Junior run #2
**# of amplicons**	123	75
**# of patients**	48	39
**# sequence reads**	77,071	118,295
**# sequences/MID**	1,602+/−1,260	3,024+/−2,203

### Success Rate of the PCR Amplification and Sequencing Method

We obtained 81 samples from HIV+ individuals with varying viral loads that ranged from undetectable-506,000 copies/ml. Altogether, we amplified all three amplicons from 58/81 (72%) of the samples we received. Using the viral load threshold of 1,000 copies/ml, which is typically used by HIV drug resistance genotyping methods developed in-house (2,000 copies/ml for FDA approved genotyping kits), we amplified 54/59 (92%) samples (viral load range = 3,280–506,000 copies/ml). Of the 5 samples above the 1,000 copies/ml viral load threshold that did not amplify all three amplicons, 1 failed to amplify the protease amplicon, 2 failed to amplify the RT1 amplicon, 1 failed to amplify the protease and RT2 amplicons, and 1 failed to amplify all three amplicons. Of the samples with PCR amplicons, 178/191 (93%) of the amplicons yielded high quality sequence data. Six protease amplicons failed to sequence, 5 RT1 amplicons failed to sequence and 2 RT2 amplicons failed to sequence. 6/13 of the failed amplicons had concentrations less than 2 ng/ul after PCR amplification and purification.

### Characterization of HIV Subtype

The sequences obtained were assembled to the HXB2 reference sequence (GenBank NC_001802) using Geneious software. A consensus sequence for each sample was exported and analyzed for subtype using the Stanford drug resistance database (http://hivdb.stanford.edu/) and the NCBI HIV subtyping tools (http://www.ncbi.nlm.nih.gov/projects/genotyping/formpage.cgi). Subtyping was confirmed through the Rega version 2.0 subtyping tool (http://dbpartners.stanford.edu/RegaSubtyping/). We found one subtype F sample, two subtype C samples, one subtype B/C recombinant, three subtype B/F recombinants and the remaining samples were subtype B. The pure subtype F sample, three B/F recombinant samples and the B/C recombinant sample amplified and sequenced all three amplicons. One sample with subtype C sequence failed to amplify the protease and RT2 amplicon, while the other subtype C sample failed to sequence the protease amplicon.

### Characterization of HIV Drug Resistance Mutations

The data (.sff file) generated at the end of the Roche/454 GS Junior run were imported into the viral sequence analysis pipeline using the parameters described in the materials and methods. Mutations were characterized as drug resistant if they matched the list of major drug resistance mutations in the Stanford HIVDB resistance matrices. For the purposes of this analysis, additional, accessory, and polymorphic mutations were not considered except for mutations found at position V179. These additional mutations typically play a more minor role in acquisition of drug resistance and are also not included in the WHO list of mutations to survey for transmitted drug resistance. Mutations at the V179 site in reverse transcriptase were part of the major drug resistance matrix until January 25, 2012, when they were reclassified as minor mutations. However, V179 mutations were found in 4/7 individuals eventually failing their drug regimen in our cohort. Overall, drug resistance mutations were identified in 13/70 (18.5%) of patient samples with sequence information (Table 3). 20% (10/50) of drug naïve samples harbored drug resistance mutations (without considering V179 mutations, the frequency of mutations in drug naïve samples is 12% (6/50)). 60% (3/5) of treatment failure samples with viral loads >1,000 copies/ml and with full sequence coverage exhibited drug resistance, while 17% (1/6) of treatment success patients with partial or complete sequence coverage (all with undetectable viral loads) harbored drug resistance mutations. Overall, 50% (6/12) of individuals failing treatment with some or full sequence coverage irrespective of viral load exhibited drug resistance mutations before and/or after treatment. The number of individuals on treatment that were sequenced in this study is very small and therefore frequencies of drug resistance presented here in treatment failures are not generalizable to the population in Brazil.

**Table 3:** Drug resistance mutations identified in the patient samples sequenced using the Roche/454 GS Junior HIV drug resistance genotyping method.

Sample ID	Time relative to ART	Resistance mutation	Drug class	Frequency (%)	Adjusted coverage	Failed Rx?
**1001**	Before	K103N	NNRTI	98.4	62	
	After	N/A				No
**1083**	Before	G190A	NNRTI	99.5	2019	
		Y181C	NNRTI	99.5	1913	
	After	N/A (Died)				Yes
**1053**	Before	K103N	NNRTI	100.0	179	
	After	N/A				No
**2018**	Before	K103N	NNRTI	99.6	253	
		E138G	NNRTI	45.8	1348	
	After	N/A				No
**2015**	Before	M46I	PI	4.7	300	
	After	N/A				No
**1050**	Before	M46I	PI	98.1	107	
		I54V	PI	100.0	204	
		V82S	PI	100.0	205	
		L210W	NRTI	98.3	2290	
		T215Y	NRTI	84.3	1612	
		M41L	NRTI	99.3	150	
		D67N	NRTI	24.1	83	
	After	M46I	PI	99.2	515	No
		I54V	PI	99.6	1146	
		V82S	PI	99.8	1150	
		L210W	NRTI	99.6	978	
		T215Y	NRTI	78.0	615	
		M41L	NRTI	98.0	49	
		K65R	NRTI	11.1	9	
		D67N	NRTI	13.5	37	
**1128**	Before	K103N	NNRTI	23.4	124	
	After	N/A				N/A
**2031**	Before	V179E	NNRTI	99.8	988	
	After	M184V	NRTI	99.7	641	Yes
		V179E	NNRTI	99.3	587	
		K103N	NNRTI	85.7	42	
**1080**	Before	V179D	NNRTI	17.7	767	
	After	V179D	NNRTI	99.5	375	Yes
**1094**	Before	None				
	After	V179D	NNRTI	11.8	722	Yes
		K103N	NNRTI	13.9	36	
**2032**	Before	V179E	NNRTI	99.2	499	
	After	N/A				Yes
**1004**	Before	None				
	After	K103N	NNRTI	77.2	22	Yes
**1033**	Before	None				
	After	V106M	NNRTI	6.5	170	Yes

The two most common drug resistance mutations found prior to treatment were V179D/E and K103N. These mutations are associated with resistance to efavirenz, a NNRTI component of first-line regimens in Brazil. All patients exhibiting V179 mutations prior to drug treatment subsequently failed drug treatment. Conversely, K103N mutations found prior to treatment never resulted in drug failure, but were found in three individuals after failing treatment. Overall, prior to treatment, eight patients had resistance to NNRTIs only, one patient had resistance a protease inhibitor (PI) only, and one patient showed resistance to a PI + NRTI (Table 3). Three of the drug naive patients with drug resistance mutations (patient IDs 1083, 1053, and 2032) were sequenced from samples at the time of diagnosis (visit 1). These mutations may therefore exemplify transmitted drug resistance. The remaining six treatment naïve patients with drug resistance mutations were sampled at different time points after diagnosis that ranged from visit 2 to visit 9 and therefore there is less certainty about whether these mutations were transmitted or a result of spontaneous mutation. 53% (7/13) of the samples harboring drug resistance mutations either before or after treatment, failed their treatment regimen.

## Discussion

We have described a method to multiplex together 48 patient samples to simultaneously test for HIV drug resistance using Roche/454 pyrosequencing on the GS Junior. We tested this method on 81 patient samples collected in São Paulo, Brazil. Most notably, we identified mutations associated with drug resistance in 20% of treatment naïve patients in our cohort. Importantly, the most frequent drug resistance mutations identified (K103N and V179D/E) correlate with drug resistance to efavirenz, a commonly used drug in first-line therapy in Brazil and many other countries. In addition, K103N, V179D/E, and other NNRTI mutations have a low genetic barrier whereby a single mutation can render resistance. This finding emphasizes the importance of surveying for transmitted HIV drug resistance and highlights the significance of developing tools such as those provided here to perform these surveys faster and cheaper to choose optimal first-line therapies.

The results presented here are similar to those previously reported, showing a frequency range of 6.5%–26.9% for treatment-naïve individuals from Brazil harboring HIV drug resistance mutations [8], [12]–[14]. Sanabani et al. reported a 13.7% frequency (n = 101) of primary drug resistance based on the WHO list of mutations for surveillance of transmitted drug resistance from the same Brazilian cohort reported in this study using traditional Sanger-based genotyping of proviral DNA [8]. Similarly, we found that 12% of drug naïve individuals from the same cohort harbored drug resistance mutations when considering only the WHO list of mutations (this excludes V179D and V179E mutations). Therefore, our sequencing method yielded similar primary drug resistance frequencies as Sanger-based methods performed on the same Brazilian cohort.

It should also be noted that there were 33 treatment-naïve patient samples used in our cohort that were previously genotyped for drug resistance using Sanger-based methods on proviral DNA [8]. Many of these previously published patient samples were from the first time point of diagnosis while those in our cohort were often from time points beyond the first visit. There was concordance of drug resistance mutations between the two studies with two exceptions. The first exception was one sample (patient ID 2018) where Sanabani et al. found a M184V mutation at visit 1 that was detected in only 1 sequence by pyrosequencing in the visit 5 sample we pyrosequenced. It is likely that during the time between visit 1 and 5 this mutation waned due to the absence of selection pressure by antiretroviral drugs. The second exception was patient ID 2015 where our method picked up a M46I protease mutation at a frequency of 4.7%, well under the frequency limitation of Sanger-based methods. Patient 2015 was the only sample with low frequency mutations found by our method before treatment that was also sequenced by the Sanabani et al., but suggests that our method will detect mutations at a lower frequency than the Sanger-based genotyping methods.

It is clear that viral load significantly impacted PCR amplification of samples in our cohort. By designating a minimum viral load of 1,000 copies/ml, we improved the frequency of successful amplification of all three amplicons from 72% to 92%. It is possible that some samples that failed to amplify are from non-B subtypes or recombinants, which requires additional optimization of PCR primers. Many samples with viral loads >1,000 copies/ml that did not amplify all three regions of Pol did amplify 1 or 2 regions. This inability to amplify all three amplicons likely stems from mismatches between the PCR primers and the HIV template, which can occur between different viruses in the same subtype. As sequencing technology advances and read lengths continue to improve, it may become feasible to generate and sequence a single amplicon spanning the entire drug resistance region in Pol (∼1,000 nt) that would further improve the efficiency of this method. The current Roche/454 titanium chemistry is limited to sequence lengths of about 450 bp. This chemistry will be the only available chemistry on the laboratory-scale Roche/454 GS Junior, however, longer read lengths may be feasible with advanced Roche/454 chemistries being developed on their full-size Roche/454 GS FLX pyrosequencer. As exemplified by the 99% success rate of initial amplification seen with a single primer pair in the FDA approved ViroSeq Sanger-based drug resistance genotyping assay, the fewer primers required to amplify HIV, the less likely mismatches between the primer and template result in inability to PCR amplify HIV [15]. The larger concern with the ViroSeq method occurs downstream during sequencing when seven primer pairs are required to match the HIV template for the sequencing reactions.

The >1,000 copies/ml viral load requirement designated for this method is the same as the those required for the ViroSeq genotyping kit as well as in-house genotyping options, ranging from 1,000–2,000 copies/ml. In addition, WHO standards to survey for emergent drug resistance mutations suggest HIV drug resistance genotyping be performed only on samples with viral loads over 1,000 copies/ml [16]. Since most samples tested for HIV drug resistance would either include individuals not yet on treatment for surveillance of transmitted drug resistance, or individuals failing treatment, a viral load threshold of 1,000 copies/ml is reasonable.

It is difficult to compare the results from Roche/454 pyrosequencing to methods currently using Sanger-based approaches because they use completely different sequencing chemistries. One published assessment of the ViroSeq assay showed that while 99% of their initial PCR amplifications were successful, success of full bidirectional sequencing using each of their seven primers varied from 56% to 100% in samples from different subtypes [15]. This range in successful sequencing is likely due to possible mismatches between the seven sequencing primers and the HIV template. This problem is eliminated in pyrosequencing because the sequencing primer binds to an adaptor sequence that is engineered into the PCR primers used to prepare each sample for sequencing.

The ViroSeq assay was specifically designed to test HIV subtype B samples and indeed these samples yielded the highest success rate for the ViroSeq assay. In one study, 6/7 of the primers sequenced the subtype B template in 100% of the 12 samples tested and 1/7 primers sequenced 83% (10/12) of subtype B samples [15]. However, for subtypes F and C, 78% and 69% of samples yielded bidirectional sequence coverage using Viroseq. In our assay, also designed specifically around subtype B, we obtained high quality sequences from 93% of the amplicons included in our sequencing pools. Our samples were primarily subtype B, but also included a few subtype F and C samples as well as recombinants. The overall efficacy of this method for non-B subtypes has not been thoroughly tested yet, however, we have redesigned some of the primers based on subtype C sequence and have been able to amplify subtype C reference viruses with these primer sets. Reasons for sequence failure in pyrosequencing are not due to primer mismatch with template, like in Sanger sequencing, but may be related to issues during the pooling process. This is further supported by the fact that there is no pattern of a particular amplicon sequencing less often than another, suggesting that it is not a sequence-specific problem. Instead, low concentrations of the amplicon DNA after purification may lead to less reliable quantitation and pooling or poor amplification of a specific product during emPCR. Some of the sample pooling done in this study was performed by an automated liquid handler, therefore it is possible that some samples were unintentionally left out of the pool if their volumes were at the minimum required for the robot and no volume was picked up out of those wells and added to the pool. Given the chemistry of pyrosequencing, there are few reasons for sequencing failures once a high quality amplicon is produced during the initial PCR amplification and after proper purification of that PCR product. More robust pooling options as well as setting a threshold for minimal concentration of a PCR purified product required for this assay may improve our 93% success rate of sequencing those amplicons included in our pool.

One of the main advantages of ultra-wide drug resistance genotyping is the economy of scale savings associated with dividing a sequencing run between 48 samples. While the cost of preparing the samples for sequencing is comparable between in-house genotyping, commercial-based kits like TruGene or Viroseq, and 454 pyrosequencing, the cost of sequencing alone is about 3–5 times less per sample for Roche/454 GS Junior sequencing over traditional Sanger-based sequencing. A Roche/454 GS Junior run costs about $1000 or ∼$20 per sample when 48 samples are multiplexed together. This is even less than previous publications utilizing the full-size Roche/454 GS FLX sequencer for drug resistance surveillance, which costs approximately $57/sample when 48 samples are run in two 1/16 lanes [17]. Therefore, it is possible to leverage the tremendous savings of pyrosequencing and scale of this method to improve feasibility of drug resistance surveillance. In addition, our pyrosequencing method can sequence 48 patient samples at one time yielding hundreds of sequences per sample while traditional HIV drug resistance genotyping can be performed on 12 samples at a time yielding 2 sequences/sample.

The release of laboratory-scale and affordable next-generation sequencers like the Roche/454 GS Junior also improves access to this technology in resource-limited settings where drug resistance surveillance is most necessary. This benchtop sequencer can allow in-country genotyping to reduce cost and has the advantage that fewer samples are required to fill a run than a full size machine (384 samples). This allows more frequent genotyping and may be able to accommodate clinical genotyping once validations and quality control mechanisms are in place. Though our use of the Roche/454 GS Junior may be higher than could be expected in a resource-limited setting, in 2011 we ran 250 sequencing runs in a mid-sized academic research laboratory. Thus, even on this benchtop sequencer, many samples can be accommodated and given the WHO recommendation of sequencing at least 47 samples for transmitted drug resistance surveillance in a given region, one GS Junior may be sufficient for an entire country or even a region spanning multiple countries [18]. On the other hand, in places like South Africa where thousands of individuals may require drug resistance testing for surveillance or clinical care, this method could easily be scaled-up using the full-sized Roche/454 Titanium FLX system along with robot-based vRNA isolations and PCR amplification set-up to accommodate an estimated 384 patient samples/run. Lastly, since very little starting material is required for pyrosequencing, it may be possible to take leftover viral RNA samples from viral load assays and RT-PCR amplify those products for pyrosequencing. In fact, one study was recently published showing that dried blood spots could be used for drug resistance genotyping of 48 patients samples pooled together by Roche/454 GS FLX sequencing [17].

We emphasize the simplicity of the data analysis pipeline used to find drug resistance mutations from our pyrosequencing data. We analyzed sequences using a custom viral sequencing analysis pipeline that allows simple upload of the .sff file generated at the end of the sequence run, selection of the optimal parameters (aligner to use, quality scores to use. etc.) through a graphical user interface, and generation of a drug resistance table at the end of the pipeline that can be integrated into a database. The drug resistance table shows all drug resistance amino acid mutations, their frequency, number of sequences representing that site and number of poor sequences that were masked during the pipeline. The analysis process for a pool of 48 patient samples was completed in approximately 1 hour. In addition, reports are generated for each sample where all SNPs and/or amino acid mutations can be interrogated (not just drug resistance data), visualized, and exported for further analysis. One advantage of this sequencing pipeline is the ability to call SNPs in phase. This means that when two nucleotides within a codon in the same sequence read are changed, that information is stored and translated together correctly. Most other analysis pipelines we tested interrogate all SNPs without considering phase and assume the other two nucleotides match the reference sequence when translating the SNP into an amino acid, which can result in incorrect translations. This data analysis pipeline is available as a module for LabKey Server, an open-source platform for the management of scientific data.

There have been several publications describing the use of next-generation sequencing to study extremely minor drug resistance mutations in an ultra-deep manner in single individuals [19]–[21]. However, while there has been two previously published manuscripts describing the use of pyrosequencing on pooled samples for HIV drug resistance genotyping, this is the first work to show the utility of this method on de novo plasma samples that can be distinguished between each other through MID tags and covers all known protease and reverse transcriptase drug resistance mutations [17], [22]. In addition, we describe and provide a simplified and open-source sequence analysis pipeline that allows accurate detection of mutations found at frequencies at least as small as 5%. Lastly, utilization of the lab-scaled Roche/454 GS Junior over the full size Roche/454 GS FLX has the added advantage of lower cost implementation in limited resource settings (6-fold cheaper) as well as reduced costs per sample (3-fold cheaper). Sequencing technology is improving at rapid speed, however the Roche/454 GS Junior titanium chemistry yields the most reliable and longest read lengths at this time (∼450 bp) on a benchtop machine. These longer reads allow possible linkage between mutations found within each of the three amplicons that may be important when interpreting drug resistance data. An additional advantage of the method described here over that previously describing sequencing of pools derived from dried blood spots, is the use of bidirectional sequence coverage through tagging both the forward and reverse primer. Bidirectional sequence coverage improves overall sequence depth of coverage per sample without adding per-run cost, which improves the accuracy of lower frequency mutations. As sequencing technology continues to advance, other lower-cost sequencing platforms like the Illumina MiSeq (read lengths at 150 bp), which do not have the same problems with homopolymer sites as pyrosequencing, may become more cost effective and easier to use.

An important consideration when using pyrosequencing techniques to assess HIV drug resistance is that there are 17 known drug resistance mutations in the protease and reverse transcriptase genes located either within or adjacent to homopolymer regions in the HXB2 backbone (Table 4). This is because homopolymers can result in stalling of reverse transcriptase, which make mutations in these areas more likely during HIV replication or during in vitro cDNA synthesis [23]. As mentioned earlier, homopolymer sequence regions present an inherent problem with pyrosequencing, which can result in false insertions or deletions at these sites. To address SNPs that may be present in these homopolymer regions we limited our analysis to only those sequences with high quality scores (>25) at positions reporting an insertion or deletion relative to the reference in order to minimize false nucleotide base calls due to homopolymers. While our control sequencing of a subtype B HXB2 virus indicates that false calls due to homopolymer regions in a subtype B backbone do not impact known drug resistance sites to a significant level (<0.71%), the same may not be true in the backbone of other subtypes. Therefore, each HIV subtype will require validation of the impact of homopolymer sites on drug resistance mutations identification. For example, one of the mutations present in a homopolymer region is K65R in the reverse transcriptase gene. The mutation that is required to change lysine into arginine occurs two nucleotides from the end of a homopolymer and would likely disrupt the homopolymer sufficiently to result in few miscalls, similarly to what is also found with the K103N mutation. However, in subtype C viruses, the genetic background is different in the K65R region of pol and the mutation that is required to result in an arginine change is located at the end of a homopolymer region [21]. This results in more potential miscalls through pyrosequencing as well as increased abundance of this mutation due to stalling of the reverse transcriptase at that site during HIV replication [24]. These sites will require further analysis to help automate interpretations of mutations identified in these regions using pyrosequencing technology. Limiting sequence analysis to only high quality sequences and reporting only mutations with frequencies above our error threshold (0.71%) can help prevent falsely identifying drug resistance mutations that fall within or adjacent to these homopolymer regions.

**Table 4:** Drug resistance mutations located within or adjacent to homopolymers in the HXB2 HIV subtype B reference sequence.

Drug class	Mutation	Sequence in subtype B^1^
**PI**	M46I/L	AAAA**AUG**
	I47V/A	**AUA**GGGGG
	G48V/M	**GGG**GG
	I54V/T/A/L/M	TTTT**AUC**AAA
	N88S	A**AAU**
**NNRTI**	L100I	GGG**UUA**AAAAAG
	K101E/P	UUA**AAA**AAG
	K103N/S	AAAAAAG**AAA**AAA
	M230L	GG**AUG**GG
**NRTI**	K65R	AAAG**AAA**AAAG
	D67N	AAAAAA**GAC**
	T69ins	**ACU**AAA
	K70R	CU**AAA**UGG
	L74V	AAAA**UUA**G
	Y115F	**UAU**UUUU
	Q151M	**CAG**GG
	K219Q/E	**AAA**AAAC

1 Codons representing the mutated amino acid are shown in boldface.

We chose to focus our efforts on mutations present in at least 5% of the virus population. This threshold was chosen primarily to be well above our error threshold, however, there is also conflicting evidence about whether mutations influence drug failure when they are present at levels below 5%, or even the 20% detection limit of current drug resistance genotyping methods [20], [21], [25]–[30]. Therefore we did not focus our efforts on mutations present at very low frequencies, which require additional considerations with 454 pyrosequencing data. Most of the mutations identified in this study were present at high frequencies nearing 100%. However, we detected mutations with frequencies as small as 4.7% (when limiting analysis to those at 5% or above when rounding), which would have been missed with the current HIV drug resistance genotyping method that has an accepted limit of detection at a frequency of 20%. Despite the controversy, there is some evidence that detection of mutations at frequencies below the current genotyping method limit of detection may be beneficial in HIV drug resistance surveillance efforts for early detection of transmitted drug resistance [31]. Overall, we detected 7 mutations with frequencies under 20% that would theoretically be missed by traditional Sanger-based genotyping methods, 4 of which occurred in 3 patients failing treatment (Table 3).

Overall, our approach produces high quality sequence data that is more sensitive and less expensive than the currently available HIV drug resistance genotyping methods. We are currently seeking ways to improve the percentages of successful amplification and sequencing using this method. Until these improvements have been made, this method would likely be best utilized for drug resistance surveillance efforts. We believe this approach can be optimized to accurately genotype HIV from different subtypes and can be scaled up to accommodate extensive efforts for drug resistance surveillance currently proposed in many low and middle-income countries. Lastly, with proper validations, this method could improve accessibility of HIV drug resistance genotyping to patients in low-income settings to improve clinical care and success of antiretroviral therapy.

## Supporting Information

Text S1 **The error associated with our HIV drug resistance method outside of drug resistance sites.** This text describes the expected error frequency associated with RT-PCR, pyrosequencing, and analysis of sequence information outside of the specific mutations associated with drug resistance. Due to sites of homopolymers, the error rate outside of drug resistance mutations is higher than within drug resistance sites.
(DOCX)

Table S1 Primer sequences for three amplicons spanning the HIV pol gene. Amplicon name (Pro, RT1, or RT2), primer name, MID tag, and primer sequence for 48 different forward and reverse primers for each of the Pro, RT1 and RT2 amplicons are provided.
(XLSX)

Table S2 Single nucleotide polymorphisms identified in either a clonal HIV viral stock or plasmid. Polymorphisms found either outside of drug resistance sites or that do not generate a drug resistance mutation are presented if found at a frequency >5% within the clonal HIV viral stock or plasmid sequences following RT-PCR amplification and Roche/454 pyrosequencing. These polymorphisms are considered error due to the method because the viral stock and plasmid should have no polymorphisms relative to the reference sequence used to compare with our samples.
(DOCX)
