# Supplementary material for: Low-Cost Ultra-Wide Genotyping Using Roche/454 Pyrosequencing for Surveillance of HIV Drug Resistance
Source: PLoS One. 2012 May 4;7(5):e36494. doi: 10.1371/journal.pone.0036494 (PMC3344889; doi:10.1371/journal.pone.0036494)
Supplement: Text S1 — The error associated with our HIV drug resistance method outside of drug resistance sites. This text describes the expected error frequency associated with RT-PCR, pyrosequencing, and analysis of sequence information outside of the specific mutations associated with drug resistance. Due to sites of homopolymers, the error rate outside of drug resistance mutations is higher than within drug resistance sites. (DOCX) [file pone.0036494.s001.docx]

**Supporting Information Text S1**

Assessment of the regions outside of the drug resistance sites or in changes that don’t create specific drug resistance mutations, show a total of 7 nucleotide mutations and 1 insertion that were present at frequencies above 5% in our clonal viral stock and/or plasmid (Table S2). These seven mutations are assumed to be the result of PCR, pyrosequencing, or alignment errors. In fact, all SNPs were found at the beginning or end of a homopolymer stretch and are likely the direct result of false insertions/deletions (indels), a known error associated with pyrosequencing. False indels can lead to incorrect nucleotide substitutions when they are not handled appropriately by an aligner during translation, compounding the difficulty with handling these pyrosequencing errors. Based on this data, if considering any SNPs outside of drug resistance sites using the analysis described here, a minimum frequency requirement of 20% should be applied to any analysis to ensure the mutation is not associated with error in the method. However, by increasing the minimum quality scores associated with SNPs and indels from 25 to 35 during the analysis pipeline, no “false” SNPs or indels are found in the control sequencing outside or inside of drug resistance regions above a frequency of 1.0%. Using a quality score stringency of 35, however, also reduces the number of true drug resistance mutations that are found in the patient samples. Since the error rate within drug resistance sites is very low (<0.7%) using the minimum quality score of 19 for SNPs and 25 for insertions/deletions, this was used to analyze the patient samples to maximize the detection of drug resistance mutations and minimize the error associated with the method.
