# Supplementary material for: Low-Cost Ultra-Wide Genotyping Using Roche/454 Pyrosequencing for Surveillance of HIV Drug Resistance
Source: PLoS One. 2012 May 4;7(5):e36494. doi: 10.1371/journal.pone.0036494 (PMC3344889; doi:10.1371/journal.pone.0036494)
Supplement: Table S2 — Single nucleotide polymorphisms identified in either a clonal HIV viral stock or plasmid. Polymorphisms found either outside of drug resistance sites or that do not generate a drug resistance mutation are presented if found at a frequency >5% within the clonal HIV viral stock or plasmid sequences following RT-PCR amplification and Roche/454 pyrosequencing. These polymorphisms are considered error due to the method because the viral stock and plasmid should have no polymorphisms relative to the reference sequence used to compare with our samples. (DOCX) [file pone.0036494.s003.docx]

**Table S2. Single nucleotide polymorphisms identified in either a clonal HIV viral stock or plasmid that were sequenced using our drug resistance genotyping method.**

| Nt position | Mutation | vRNA plasmid | % frequency | Adj. # of seqs.^1^ | Total Ns^2^ | Found in Sanger? |
| --- | --- | --- | --- | --- | --- | --- |
| 1849 | G->C | vRNA | 5.6 | 606 | 131 | No |
|  |  | Plasmid 1 | 2.7 | 489 | 232 |  |
|  |  | Plasmid 2 | 2.5 | 727 | 379 |  |
| 2181 | A->G | vRNA | 2.9 | 246 | 452 | No |
|  |  | Plasmid 1 | 3.8 | 287 | 292 |  |
|  |  | Plasmid 2 | 13.6 | 346 | 563 |  |
| 2395 | A->T | vRNA | 18.5 | 27 | 693 | No |
|  |  | Plasmid 2 | 16.7 | 54 | 1097 |  |
| 2400 | A->G | vRNA | 12.0 | 25 | 694 | No |
|  |  | Plasmid 1 | 6.1 | 31 | 665 |  |
|  |  | Plasmid 2 | 15.8 | 38 | 1110 |  |
| 2402 | A->G | vRNA | 4.7 | 85 | 634 | No |
|  |  | Plasmid 1 | 5.0 | 119 | 577 |  |
|  |  | Plasmid 2 | 3.0 | 332 | 815 |  |
| 2407 | A->T | vRNA | 16.9 | 166 | 553 | No |
|  |  | Plasmid 1 | 1.2 | 246 | 450 |  |
| 2482 | _->T | Plasmid 1 | 9.1 | 1538 | 25 | No |
|  |  | Plasmid 2 | 7.5 | 1668 | 16 |  |
| 2750 | A->C | vRNA | 9.0 | 144 | 675 | No |

^1^ Adj.# of seqs. is the number of sequences representing that nucleotide position after removing low quality sequences.

^2^ Total Ns refers to the number of sequences containing a poor quality nucleotide at that nucleotide position. These are eliminated in the Adj. # of seqs value.
